# Correlation between Subjective Nasal Patency and Nasal Capacity in Young Adults: A Pilot Study with a Prototype Device—A Nasoorospirometer

**DOI:** 10.3390/jcm13092506

**Published:** 2024-04-24

**Authors:** Katarzyna Zasadzińska-Stempniak, Hanna Zajączkiewicz, Andrzej Kukwa

**Affiliations:** Department of Otorhinolaryngology, Head and Neck Diseases, School of Medicine, University of Warmia and Mazury in Olsztyn, al. Warszawska 30, 10-082 Olsztyn, Poland

**Keywords:** sleep apnea syndrome, sleep health, obstructive sleep apnea, screening, sleep study, young adults, disease severity

## Abstract

**Background:** Nasal airway obstruction (NAO) is characterised by high resistance in the nasal cavity with a collapsible and narrowed upper airway and is an integral part of OSA pathophysiology. The literature demonstrates that the identification of high-risk OSA in the young adult population leads to the prevention of later health consequences. A nasoorospirometer is a prototype device that measures nasal capacity during inspiration. The basis for measurement is a Wheatstone bridge and a thermal anemometer. The parameters are recorded via hot wire anemometry (HTA) with velocity measurements in the airflow field. Therefore, this pilot study aimed to test the feasibility of the device by examining a young adult sample. The secondary aim was to determine whether subjective NAO correlates with nasal capacity and whether NAO corresponds with anthropometric parameters and individual risk of OSA. **Methods**: A group of 31 participants (mean age 24.9 years) underwent a thorough laryngological examination. The nasoorospirometer was used to measure objective NAO (nasal capacity), the NOSE scale was used to gain subjective NAO evaluation, and the Berlin Questionnaire for the risk of OSA. **Results**: A correlation analysis confirmed no significant associations between the subjective and objective measures (*p* > 0.05). Higher BMI and neck circumference are associated with lower NAO and higher nasal patency in the population of young adults (r: 0.32–0.45; *p* < 0.05). The risk of OSA showed no statistically significant association (*p* > 0.05). **Conclusions**: We presented three methods of NAO assessment: subjective participant evaluation, objective nasoosopirometry, and objective laryngological assessment. However, the use of a nasoorospirometer with anthropometric measures in young adults needs to be verified in future studies.

## 1. Introduction

Obstructive sleep apnea (OSA) is a condition primarily associated with the narrowing of the upper airways, which is characterized by repeated episodes of total or partial pharyngeal occlusion during sleep. Moreover, high resistance in the nasal cavity with a collapsible and narrowed upper airway is the typical feature of nasal airway obstruction (NAO) [1,2]. Various degrees of NAO and increased nasal resistance often accompany OSA and increase the risk of snoring [3,4,5,6,7]. Further, isolated nasal surgery can possibly improve OSA subjectively, especially in patients with NAO, or improve treatment effectiveness with continuous positive airway pressure (CPAP) [8,9,10]. Other factors that increase the risk of OSA are high body-mass index (BMI) and increased neck circumference (NC). Unfortunately, data on the association of NAO with BMI and NC are scarce. Only a few studies have compared the occurrence of NAO in different body weight status groups and explored the association between anthropometric measures and NAO occurrence [11,12,13]. The literature demonstrates that the identification of a high risk of OSA in the young adult population leads to the prevention of later health consequences [14,15]. This age group may have an unconscious nasal patency disorder that increases the risk of OSA. In the case of early OSA risk detection, it is possible to prevent the consequences to which developed OSA leads, e.g., stroke, pulmonary hypertension, and atrial fibrillation [16,17,18].

Due to the low availability of full polysomnography (PSG) studies, simplified strategies to diagnose or suspect OSA are being proposed, among them non-contact biomotion sensors, and sleep switch devices [19]. Since NAO is a frequent condition in OSA, its assessment can indicate a possible sleep disorder. By examining NAO parameters, e.g., nasal capacity, we could determine a predisposition to OSA. The addition of an NAO assessment to anthropometric measures can provide relevant information for OSA prediction and may perhaps become a screening test for the risk of OSA.

This study aimed to test the feasibility of the prototype device, the nasoorospirometer, to refine the technique and identify flaws. The secondary aims were to analyse nasal capacity in young adults and investigate whether subjective NAO correlates with nasal capacity. Moreover, we aimed to evaluate whether NAO correlates with anthropometric parameters and the risk of OSA. We hypothesized that subjective and objective nasal obstruction do not correlate with each other.

## 2. Materials and Methods

### 2.1. Study Design

We conducted a pilot cross-sectional study. Our study recruitment comprised 31 students and employees recruited from the University of Warmia and Mazury in Olsztyn, Poland, in 2022.

Subjects were included if they filled the following criteria:

(1) aged between 18–30 years;
(2) gave their written informed consent for the examination with a nasoorospirometer prototype device.

The exclusion criteria were:

(1) the active upper respiratory tract infection;
(2) history of neoplastic or autoimmune processes;
(3) uncontrolled chronic disease;
(4) current pregnancy.

Ethical approval was obtained from the ethics committee of the University of Warmia and Mazury (reference number 23/2020). All participants gave written informed consent.

Baseline characteristics of the participants (age, gender, weight, height, and neck circumference) and prior medical history were collected from the participants using a standard questionnaire.

Participants filled out the Nasal Obstruction Symptom Evaluation (NOSE) score to collect information on their subjective symptoms. This is a five-item scale in which an individual participant reports over the past month if he or she reports the symptoms of nasal congestion, nasal obstruction, difficulty breathing through the nose, difficulty sleeping, and sensation of air hunger using a scale from 0 (no problem) to 4 (severe problem). The numbers are summed up and multiplied by 5 to get a score ranging from 0 (no) symptoms to 100 (severe) symptoms [15].

A laryngological examination with otoscopy, nasal endoscopy, and laryngoscopy was conducted according to an established scheme before the examination with a nasoorospirometer. The examination was carried out in all subjects by the same otorhinolaryngologist with 10 years of clinical experience. The presence of anatomic nasal airway obstruction (deviated septum, medically resistant turbinate hypertrophy, or nasal valve dysfunction) was determined. Data were obtained at the same time to decrease the influence of the nasal cycle. The study was conducted in a controlled constant-temperature room to minimize the effect of the surroundings on the nasal mucosa.

The neck circumference was measured at the mid-neck, between the midline of the cervical spine and the midline of the neck, with the participant standing upright and facing forward, with arms relaxed. The circumference was measured just below the protrusion in men with a laryngeal protrusion [19].

The Berlin questionnaire (BQ) was administered to determine risk factors for obstructive sleep apnea (OSA). This questionnaire contains 10 questions on the following three categories: snoring (category 1, items 1–5), daytime somnolence (category 2, items 6–9), and presence of obesity or hypertension (category 3, item 10). High risk in at least two categories is considered high risk for OSA [14].

### 2.2. Nasoorospirometer

The nasoorospirometer provides the following objective measurements of NAO: unilateral and total inhale number, mean capacity, duration of the inhalation, and maximum and minimum capacity. We analysed the aforementioned parameters during inspiration. The same technician examined each subject for 2 min and read the data.

Both nostrils were tested simultaneously. A single measurement takes about a few seconds, and the entire testing process is completed in two minutes. After powering on the device and starting the computer program, calibration is carried out simply by complying with the on-screen instructions. The calibration should be performed each time the device is opened. The participant should be seated and not change position during the test. Swallowing should be avoided during measurements. The participant was instructed to breathe casually. To obtain a natural breathing pattern, participants were asked to scroll the Internet.

**Figure 1:**
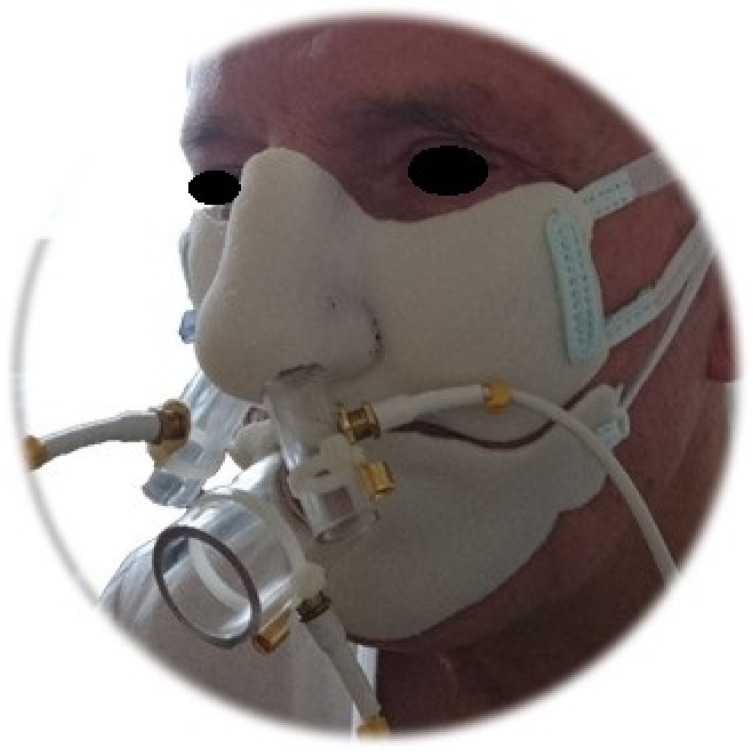
Placement of the printed mask with sensors on the patient’s face.

**Figure 2:**
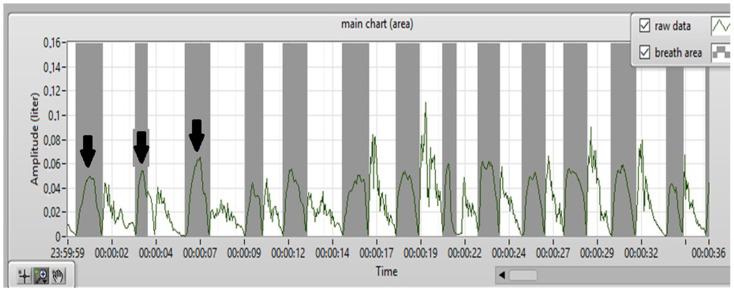
An example cutout of the result obtained from the device. The figure shows a measurement of nasal capacity from one nostril as a function of time. Vertical arrows indicate inspiration.

Figure 1 presents the placement of the mask with the sensors on the participant’s face. The device is composed of a face mask, a central processing unit, and a computer. The oronasal mask is divided into sealed nasal and oral compartments with sensors: two nasal and one oral. The basis of data acquisition is three sensors that detect the nasal airflow and alterations in the turbulence kinetic energy transfer. Our group described our prototype device’s principles of operation and configuration [20]. An example of the result from the measurements is shown in Figure 2.

Each orifice has a sensor that detects parameters: inhale/exhale number, capacity, and breath length. Two independent sensors connected to a differential pressure transducer and an amplifier were attached to the nasal and oral compartments. The basis for measurement is a Wheatstone bridge and thermal anemometer. The parameters are recorded via hot wire anemometry (HTA) with velocity measurements in the flow field. Applying HTA allows for the calculation of instantaneous flow velocity based on electrical voltage measurements. The nasal airflow alters turbulent kinetic energy, which is the basis of data acquisition. Airflow data from the nasoorospirometer were acquired using LabVIEW (National Instruments, Texas, UT, USA) and synchronized with the system.

### 2.3. Statistical Analysis

The study characteristics were summarized using mean and standard deviation for continuous traits and number and percentage for categorical traits. The correlation between objective, subjective, and anthropometric measures was assessed using Spearman’s correlation coefficients. Differences in objective nasal patency between groups with a low and a high risk of OSA were assessed using the Mann–Whitney U test. A two-sided *p* < 0.05 was considered statistically significant. All analyses were conducted in R (version 4.1.0, R Foundation for Statistical Computing, Vienna, Austria).

## 3. Results

The baseline characteristics of study participants are summarised in Table 1. Among the 31 participants included in this study, 13 were female, and 18 were male. The mean age of the participants was 24.9 (SD: 2.6) years. The mean BMI of the participants was 23.7 (SD: 4.5) kg/m^2^. The mean NC was 35.3 (SD: 3.9) cm. Five participants had a high risk of OSA in BQ; the rest had a low risk. Six participants had severe symptoms according to the NOSE score, nine moderate, and 14 mild. Two had no symptoms, while none of the participants had extreme symptoms. Sixteen participants had nasal obstruction according to the examining laryngologist.

Correlation coefficients between objective nasal patency measures obtained with nasoorospirometry and subjective nasal patency are presented in Table 2. Mean capacity showed a moderate-to-high correlation with mean capacity variability, mean time, minimum, maximum, and sum capacity (r: 0.51–0.88). Mean capacity variability was inversely correlated with inhale number (r: −0.59) and positively with the mean time, its variability, and maximum capacity (r: 0.54–0.73). None of the nasoorospirometry parameters showed a significant correlation with the subjective NOSE score (*p* > 0.05).

Body mass index showed a significant correlation (Table 3) only with mean time (r: 0.40). In contrast, neck circumference showed a moderate-to-high positive correlation with mean, maximum, minimum, and sum capacity (r: 0.32–0.45).

Table 4 presents a comparison of nasoorospirometry parameters between groups with a low and high risk of OSA. None of the parameters showed a statistically significant difference (*p* > 0.05).

## 4. Discussion

In this study, we used an innovative method of nasal airway obstruction assessment. We investigated the association between anthropometric parameters (neck circumference and BMI) and nasal capacity. We found that there is no evidence for the correlation between subjective and objective nasal patency. In addition, we showed that higher BMI and neck circumference are associated with lower NAO and higher nasal patency in the population of young adults.

We may evaluate nasal airway obstruction with subjective and objective methods. Among the former, there are different types of questionnaires, among others, the NOSE score. A golden standard tool for objective NAO measurements would be a simple, cheap, non-invasive, and reproducible method. The most accepted and readily available are rhinomanometry, acoustic rhinometry, and peak inspiratory nasal flow [21,22,23]. Computational fluid dynamics [15,24] and acoustic analysis of breathing signals [25] are distinguished among the recently developed, with promising evidence of the ability to identify the actual anatomical point responsible for the patient’s perceived nasal obstruction. We invented a prototype modular device, a nasoorospirometer, designed for nasal breathing analysis [20].

The objective assessment of nasal patency has long been a topic that remains evolving despite many possible modalities. This is because the obtained findings of the accurate nasal patency assessment often do not correspond to the patient’s subjective patency [26,27,28,29]. In addition, examinations performed using different methods may not be comparable, and reproducibility rates may differ between clinical centres [27,28,30]. However, an accurate patency assessment may be beneficial for several reasons, including grading of patency impairment, treatment design, and evaluation of patency therapeutic outcomes.

Our result confirming that the perception of NAO does not correlate with objective measurements is in accordance with another study on the subjective perception of nasal patency [27,31]. The perception of a patent nose is the result of a number of variables, such as the cooling of the air [32,33] and nasal anatomical (e.g., septal deviation) and mucosal causes (e.g., the nasal cycle of the inferior turbinate, sinusitis, rhinorrhea) [24,34]. Our study was conducted in a controlled constant-temperature room to determine the effect of the surroundings on the nasal mucosa. Andre et al. published a systematic review, analysing 16 studies on this subject, concluding that the correlation between objective and subjective sensation remains uncertain [27]. The presence or absence of correlation was equally distributed among all the 16 analysed studies and did not depend on the study’s level of evidence. However, the subjective assessment tool in all the studies that comprised this systematic review was either a Visual Analog Scale or non-validated questionnaires. Our study used a validated NOSE score, which is more valuable. Other studies confirmed poor correlation between subjective and objective measurements. There was no difference when the VAS or NOSE scores were applied [30,31]. Moreover, the studies that assessed subjective unilateral perception were more likely to have a correlation with objective measurements [24,27]. Although we assessed nostrils bilaterally, future studies might focus on a separate nostril examination.

The results of the studies analysing the association between anthropometric measures and NAO assessment are ambiguous. Our study found an association between higher BMI having higher bilateral mean capacity and lower NAO. Moreover, weight had a stronger correlation with mean capacity than height. This is contrary to previous research by Agarwal et al., where the rhinometric measures were reduced in obese patients with OSA [11]. On the other hand, Kemppainen et al. found no correlation between BMI and the results of rhinometry measurements. What is more, there were no significant changes in rhinometric values, even though a significant weight loss was achieved [12].

We found that NC correlates with nasal airway obstruction. It is comparable with the results of another study, where oronasal breathing was associated with BMI and NC [13]. Nascimento et al. performed a full night of in-laboratory polysomnography and assessed nasal breathing with pneumotachographs after that. This NAO assessment methodology may be similar to our nasoorospirometer study.

Contrary to the general consensus, we found that higher BMI and NC are associated with lower NAO. Our results may indicate that NAO may be caused not only by local causes but also by external extra-nasal causes, as also shown in other studies [1,35]. Blomster et al. showed that NAO was significantly lower in OSA patients after weight loss, which may be related to adipose tissue loss in the nasal cavity as well [36]. These findings imply that an NAO should be approached as a systemic problem, not a purely site-specific one.

In our study, we objectively characterised multiple anatomic deformities that caused NAO in each patient, although they did not correlate with subjective assessment. Contrary to the literature, specific objective measurements of NAO were correlated to subjective measures of nasal obstruction [37]. Although the group in the aforementioned study was small, it may be possible to define a nasal mean capacity pattern for each abnormality type of NAO in future studies, as was already suggested previously [24].

Some limitations of this study must be acknowledged. First, the results might be challenged due to limited measurements conducted with this device. For sure, we need more studies to test the accuracy and effectiveness of the device. However, prior research has already indicated no significant correlation between subjective and classical objective nasal obstruction assessment, which makes our results reliable. Second, we used bilateral assessment, which may negatively influence the result’s reliability, so a study assessing each nostril separately should be adopted in future research. Moreover, another limitation is the lack of data necessary to calculate the minimum sample size based on previous studies. Our study sample was relatively small, but its pilot character explains this. Moreover, other studies on this subject were also on small populations [15,24,33].

In this study, the mean NOSE score of 28.4 suggests that most subjects did not experience a relevant subjective nasal obstruction. Moreover, we suspect the presence of selection bias. Despite the expectation of greater representation of OSA patients, the recruited group had a small number of such patients. Clearly, larger observational studies need to be conducted to confirm our results. Adding a PSG and not only an assessment of OSA risk through a validated questionnaire may clarify the association better in future studies.

We presented three methods of NAO assessment: subjective patient evaluation, objective nasoosopirometry, and objective laryngological assessment. When used separately, they can only show part of the clinical picture, so we suggest that they should complement each other.

## 5. Conclusions

NAO is an integral part of OSA pathophysiology. The nasoorospirometer is a simple, cheap, and non-invasive method. By analysing objective NAO with anthropometric measures, NC and BMI might be effective in diagnosing OSA predisposition. Higher BMI and NC were noted to be associated with lower NAO in the population of young adults. Moreover, no evidence for the correlation between subjective and objective nasal patency was observed. The results of this pilot study are encouraging; however, the usage of NAO with anthropometric measures needs to be verified in future studies.

## Figures and Tables

**Table 1:** Sample characteristics.

Characteristic	*n* (%)
N of participants	31 (100%)
Age at recruitment (years)	24.9 (±2.57)
Gender:	
Male	18 (58%)
Female	13 (42%)
BMI (kg/m^2^)	23.72 ± 4.5
NC	35.32 (±3.94)
BQ	
High	5 (16%)
low	26 (84%)
NOSE SCORE	Mean: 28.4
Severe	6 (20%)
Moderate	9 (29%)
mild	14 (45%)
No symptoms	2 (6%)
Nasal obstruction	16 (52%)
Earlier septoplasty	2 (6%)

BMI—body mass index; NC—neck circumference; BQ—Berlin questionnaire; NOSE—nasal obstruction symptom evaluation.

**Table 2:** Spearman’s correlation coefficients between objective and subjective nasal patency.

	Mean Capacity (R + L) [l]	Mean Capacity (R + L) std [l]	Inhale Number (R + L)/min	Mean Time (R + L) [std(s)]	Mean Time (R + L) [s]	Max Capacity (R + L)	Min Capacity (R + L)	Sum Capacity (R + L)
Mean Capacity (R + L) std [l]	**0.51** ***p* = 0.003**							
Inhale number (R + L)/min	−0.35 *p* = 0.054	**−0.59** ***p* < 0.001**						
Mean time (R + L) [std(s)]	0.20 *p* = 0.27	**0.71** ***p* < 0.001**	**−0.40** ***p* < 0.026**					
Mean time (R + L) [s]	**0.64** ***p* < 0.001**	**0.54** ***p* < 0.001**	**−0.67** ***p* < 0.001**	**0.45** ***p* < 0.011**				
Max capacity (R + L)	**0.88** ***p* < 0.001**	**0.73** ***p* < 0.001**	**−0.39** ***p* < 0.028**	**0.40** ***p* < 0.024**	**0.65** ***p* < 0.001**			
Min capacity (R + L)	**0.55** ***p* < 0.001**	−0.10 *p* < 0.59	−0.20 *p* < 0.28	−0.07 *p* < 0.69	**0.38** ***p* < 0.034**	**0.42** ***p* < 0.019**		
Sum capacity (R + L)	**0.63** ***p* < 0.001**	0.07 *p* < 0.72	0.27 *p* < 0.14	−0.13 *p* < 0.5	0.16 *p* < 0.4	**0.52** ***p* < 0.003**	**0.39** ***p* < 0.03**	
NOSE score	0.18 *p* < 0.33	0.03 *p* < 0.86	−0.02 *p* < 0.93	−0.14 *p* < 0.45	0.19 *p* < 0.31	0.11 *p* < 0.56	−0.02 *p* < 0.9	−0.14 *p* < 0.46

Bolded are correlation coefficients that are statistically significant (*p* < 0.05). Max—maximum; Min—minimum; R—right; L—left; Sum—summed; std—standard deviation; min—minute.

**Table 3:** Correlation between nasoorospirometry parameters and anthropometric measures.

Measure	Body Mass Index	Neck Circumference
Mean Capacity (R + L) [l]	0.27 *p* = 0.14	**0.45** ***p*** **= 0.01**
Mean Capacity (R + L) std [l]	0.21 *p* = 0.27	0.21 *p* = 0.27
Inhale number (R + L)/min	−0.22 *p* = 0.22	−0.04 *p* = 0.83
Mean time (R + L) [std(s)]	0.07 *p* = 0.7	0.09 *p* = 0.61
Mean Time (R + L) [s]	**0.40** ***p*** **= 0.03**	0.34 *p* = 0.06
Max capacity (R + L)	0.23 *p* = 0.2	**0.44** ***p*** **= 0.014**
Min capacity (R + L)	0.19 *p* = 0.32	**0.32** ***p*** **= 0.04**
Sum capacity (R + L)	0.00 *p* = 1	**0.38** ***p*** **= 0.03**

Bolded are correlation coefficients that are statistically significant (*p* < 0.05). Max—maximum; Min—minimum; R—right; L—left; Sum—summed; std—standard deviation; min—minute.

**Table 4:** Comparison of objective nasal patency measures according to the risk in the Berlin Q.

Measure	Low Risk, *n* = 26	High Risk, *n* = 5	*p*-Value
Inhale number (R + L)	24 (13, 29)	30 (24, 37)	0.3
Mean Capacity (R + L) [l]	0.47 (0.33, 0.67)	0.43 (0.31, 0.45)	0.6
Mean Capacity (R + L) std [l]	0.15 (0.10, 0.22)	0.12 (0.08, 0.20)	0.7
Inhale number (R + L)/min	12.6 (10.4, 17.6)	14.3 (11.7, 17.7)	0.8
Mean time (R + L) [std(s)]	0.35 (0.27, 0.48)	0.36 (0.22, 0.47)	0.7
Mean Time (R + L) [s]	1.66 (1.35, 2.01)	1.64 (1.55, 1.86)	>0.9
Max capacity (R + L)	0.75 (0.58, 1.01)	0.75 (0.49, 0.75)	0.7
Min capacity (R + L)	0.16 (−0.02, 0.39)	0.17 (0.12, 0.19)	>0.9
Sum capacity (R + L)	12.4 (7.0, 16.0)	10.8 (9.2, 16.0)	0.8

Values are median and interquartile range. *p* value was derived from the Mann–Whitney U test. Max—maximum; Min—minimum; R—right; L—left; Sum—summed; std—standard deviation; min—minute.

## Data Availability

Data available on request due to restrictions, e.g., privacy or ethics. The data presented in this study are available on request from the corresponding author. The data are not publicly available due to the protection of personal data.
